# Gram-negative bloodstream infections in six German university hospitals, 2016–2020: clinical and microbiological features

**DOI:** 10.1007/s15010-024-02430-7

**Published:** 2024-11-25

**Authors:** Alexander Mischnik, Hannah Baltus, Sarah V. Walker, Michael Behnke, Beryl Primrose Gladstone, Trinad Chakraborty, Linda Falgenhauer, Petra Gastmeier, Hanna Gölz, Siri Göpel, Georg A. Häcker, Paul G. Higgins, Can Imirzalioglu, Nadja Käding, Evelyn Kramme, Silke Peter, Siegbert Rieg, Anna M. Rohde, Harald Seifert, Evelina Tacconelli, David Tobys, Janina Trauth, Maria J. G. T. Vehreschild, Kyriaki Xanthopoulou, Jan Rupp, Winfried V. Kern, the DZIF R-NET Study Group, Lena Biehl, Jochen Braun, Michael Buhl, Simone Eisenbeis, Hajo Grundmann, Catriona Hennelly, Florian Hölzl, Nathalie Jazmati, L. Kunstle, Dirk Friedrich, Azita Lengler, Dana Lenke, Luis Alberto Peña Diaz, Georg Pilarski, Susanna Proske, Judith Schmiedel, Norbert Thoma, B. Walinski, Janine Zweigner

**Affiliations:** 1 German Centre for Infection Research (DZIF), Braunschweig, Germany; 2 Division of Infectious Diseases, Department of Medicine II, University Medical Centre Freiburg, Freiburg, Germany; 3 Department of Infectious Diseases and Microbiology, University Hospital Schleswig-Holstein/Campus Lübeck, Lübeck, Germany; 4 Institute for Social Medicine and Epidemiology, University of Lübeck, Lübeck, Germany; 5 Institute for Medical Microbiology, Immunology, and Hygiene, Medical Faculty, University Hospital Cologne, Cologne, Germany; 6 Institute for Clinical Microbiology and Hospital Hygiene, RKH Regionale Kliniken Holding und Services GmbH, Hospital Ludwigsburg, Germany; 7 Institute for Hygiene and Environmental Medicine, Charité–Universitätsmedizin Berlin, corporate member of Freie Universität Berlin, Humboldt-Universität zu Berlin, and Berlin Institute of Health, Berlin, Germany; 8 Division of Infectious Diseases, Department of Internal Medicine 1, University Hospital Tübingen, Tübingen, Germany; 9 Institute of Medical Microbiology, Justus Liebig University Giessen, Giessen, Germany; 10 Institute of Hygiene and Environmental Medicine, Justus Liebig University Giessen, Giessen, Germany; 11 Institute for Medical Microbiology and Hygiene, University Medical Centre Freiburg, Freiburg, Germany; 12 Institute of Medical Microbiology and Hygiene, University of Tübingen, Tübingen, Germany; 13 Institute of Translational Research, Cologne Excellence Cluster on Cellular Stress Responses in Aging-Associated Diseases (CECAD), Faculty of Medicine, University Hospital Cologne, Cologne, Germany; 14 Department of Internal Medicine (Infectiology), Justus Liebig University Giessen, Giessen, Germany; 15 Department I of Internal Medicine, University Hospital of Cologne, Cologne, Germany; 16 Department II of Internal Medicine, Infectious Diseases, Goethe University Frankfurt, University Hospital Frankfurt, Frankfurt am Main, Germany

**Keywords:** Bloodstream infection, Escherichia coli, Klebsiella, Cephalosporin resistance, ESBL prevalence, Longitudinal survey

## Abstract

**Purpose:**

To analyze the longitudinal epidemiology and antimicrobial resistance (AMR) patterns of Gram-negative bloodstream infections (BSI) in Germany.

**Methods:**

Post-hoc analysis of prospectively documented BSI due to *Escherichia coli*, *Klebsiella* spp., *Enterobacter* spp., *Pseudomonas aeruginosa* and *Acinetobacter baumannii* from six university hospitals between 2016 and 2020. In a subanalysis 1228 episodes of BSI (*E. coli* N = 914, *Klebsiella* spp. N = 314) were analyzed for clinical endpoints and risk factors.

**Results:**

*E. coli* was the most prevalent cause of BSI, with 5412 cases, followed by *Klebsiella* spp. (2148 cases), *P. aeruginosa* (789 cases), *Enterobacter* spp. (696 cases), and *A. baumannii* (31 cases). BSI incidence rates were particularly high in haematology/oncology, with *E. coli* BSI reaching 13.9 per 1000 admissions. Most (58%) of the BSI episodes were community-acquired. A notable finding was the moderate increase of third-generation cephalosporin resistant Enterobacterales (3GCREB) for *E. coli* from 13.9% in 2016 to 14.4% in 2020 and a decrease for *Klebsiella* spp. from 16.5% in 2016 to 11.1% in 2020 corresponding to extended-spectrum betalactamase (ESBL) phenotype. In our analysis, the 3GCREB phenotype was not associated with a higher risk of death or discharge with sequelae for *E. coli* and *Klebsiella* spp.

**Conclusion:**

Our study provides longitudinal data on Gram-negative BSI in Germany on a clinical basis for the first time. These data underscores the critical need for ongoing surveillance and more pathogen-related clinical data.

**Supplementary Information:**

The online version contains supplementary material available at 10.1007/s15010-024-02430-7.

## Introduction

Bloodstream infections (BSI) remain a major cause of morbidity and mortality among infectious diseases worldwide. BSI caused by Gram-negative bacteria requires rapid initiation of appropriate antibiotic therapy and source control. *Escherichia coli* (*E. coli*) and *Klebsiella* spp. are major pathogens in community-acquired and hospital-acquired infections. *Klebsiella* spp. and *E. coli* bacteremia are frequently linked to urinary tract and intraabdominal infections, and rarely to pneumonia and other organ infections [1, 2]. *Enterobacter* spp. and *Pseudomonas aeruginosa* (*P. aeruginosa*) BSI are mostly associated with opportunistic nosocomial infections. Antibiotic therapy with a β-lactam is considered the treatment of choice in both community-onset and nosocomial BSI. However, a recent increase in the prevalence of third-generation cephalosporin-resistance has led to a use of β-lactams like carbapenems with broad activity [3]. β-lactamases have been recognized as the main cause of third-generation cephalosporin resistant Enterobacterales (3GCREB), whereas non-fermenters harbour various intrinsic and acquired resistance mechanisms. As levels of antimicrobial resistance (AMR) are further increasing, there is a general concern about increasing mortality in the elderly population. This increase is partly being driven by the emerging antibiotic resistant strains. The epidemiological burden of Gram-negative BSI and its clinical impact has not been well documented in Germany. Comparative data for Germany is primarily available from international surveillance systems such as EARS-Net which includes German Antibiotic Resistance Surveillance (ARS) data in an interactive database (https://ars.rki.de/). There is a need to better characterize and determine the clinical and microbiological epidemiology of Gram-negative BSI in the hospital and community to develop effective preventive measures.

In the present study, we investigate recent epidemiological trends and resistance profiles in Gram-negative BSI over a period of 5 years (2016–2020) in six German university hospitals. We were both interested whether frequencies and resistance phenotypes have changed over time in different clinical disciplines and whether this impacts patient outcomes.

## Material and methods

### Patients and setting

This post-hoc analysis included prospectively collected datasets from the German Centre for Infection Research (DZIF) “Multiresistant Bacteria" R-Net and the BLOOMY (BLOodstream infection due to multidrug-resistant Organisms: Multicenter studY on risk factors and clinical outcomes) [4] multicenter prospective non-interventional clinical cohort study, including patients with BSI in Intensive Care Unit/Intermediate Care Unit (ICU/IMC) and non-ICU/IMC settings, from 2016 (Q4 2016) to 2020 (Q2 2020), in which six German university hospitals participated. BSI surveillance included all monomicrobial and polymicrobial BSI with one of the Gram-negative target organisms (*E. coli*, *Klebsiella* spp., *Enterobacter* spp., *P. aeruginosa* and *A. baumannii*) in adult hospitalized patients (age ≥ 18 years) in each center (R-Net dataset). Clinical and further epidemiological data of all BSI were collected in the BLOOMY database at different times by study nurses in structured interviews for a patient history of 6 month prior to admission. BLOOMY clinical variables were collected on day-0, day-3, day-7 and weekly thereafter including symptoms (gastrointestinal, respiratory, neurological), presence of catheters, concomitant treatment (cytotoxic drugsstatics, systemic steroidscortisone, gastric acid inhibitors, monoclonal antibodies, antivirals) and the Pitt bacteremia score (PBS) [4]. The PBS is widely used to predict mortality in patients with bacteremia; moreover, it uses a simple scoring method and is applicable even in the general wards [5, 6]. A PBS of 0 to 3 is considered low risk, 4 to 7 moderate risk, and ≥ 8 high risk for increased mortality. Data from both datasets were merged and further analyzed according to patient identifiers with the statistical program R 4.1.3 [7]. Blood cultures were drawn as part of routine diagnostics in patients with clinical suspicion of BSI. Exclusion criteria included age less than 18 years and admission to ophthalmology, pediatrics and psychiatry/psychosomatics. Species identification and antimicrobial susceptibility testing were performed according to standard protocols.

### Definitions

Onset of BSI was defined as the date of the first positive blood culture drawn yielding a Gram-negative pathogen. Mode of acquisition was classified as hospital-acquired if the onset of BSI was > 48 h after admission or otherwise as community-acquired. The main or dominant focus of infection was identified as the most severe manifestation that determined the clinical management and length of treatment. A BSI was considered polymicrobial with evidence of either another target Gram-negative organism or *Staphylococcus aureus* or *Enterococcus* spp. However, the detection of a target organism and a bacterium from the normal skin flora (coagulase-negative staphylococci, corynebacteria, cutibacteria, etc.) was not considered to represent polymicrobial BSI. Resistance to third generation cephalosporins (3GCR) was considered a surrogate marker for extended-spectrum beta-lactamase (ESBL) phenotype in the case of cefotaxime MIC > 2 mg/L and/or ceftazidime > 4 mg/L according to EUCAST breakpoints (v 14.0) (www.eucast.org/clinical_breakpoints). Survival of patients was categorized as complete recovery (without the need for further therapy) and “discharge with sequelae” (need for further treatment in early rehabilitation facilities or other). The status of discharge was determined “with sequelae” (impaired) whenever a patient status was marked anything but “recovered”. Therefore, the number of patients who suffer from sequelae after BSI is determined by testing against all patients completely recovered or deceased.

### Ethics

We gained approval by the local ethics committee (approval number EA4/018/14). For the BLOOMY subanalysis only patients were included who gave their written informed consent.

### Statistics

Quantitative data are represented by means and standard deviations as well as median values or with absolute and relative frequencies. Differences between BSI were exploratively tested for statistical significance using Fisher's exact test for ordinally scaled variables and Wilcoxon test for metric variables. A significant difference was assumed at a p-value less than 0.05. The Mann Kendall Trend Test was used to analyze data collected over time for consistently increasing or decreasing trends (monotonic) in Y values. To determine factors influencing the outcome, multinomial regression analysis was performed using a selection of variables whose effect was biologically plausible. The analyses were performed with the statistical program R 4.1.3 [7].

## Results

### Overall antimicrobial phenotypic resistance

In the 5-year period, a total of 9076 patients with Gram-negative BSI were identified. *E. coli* was the most prevalent cause of BSI, with 5412 cases, followed by *Klebsiella* spp. (2148 cases), *P. aeruginosa* (789 cases), *Enterobacter* spp. (696 cases), and *A. baumannii* (31 cases). *Klebsiella* BSI were predominantly caused by *K. pneumoniae* (70%), *K. oxytoca* (21%) and other *Klebsiella* spp. and *Enterobacter* spp. were mainly *E. cloacae* complex (95%) (Suppl. Table 1). The median age of patients varied slightly across organisms, but was generally around 67–70 years. Male patients constituted the majority of patients with BSI, especially in *P. aeruginosa* cases (65.9%). Infections were most commonly of community-onset for *E. coli* (65.8%), but less so for other bacteria. The BSI were predominantly caused by a single organism, with *E. coli* showing the highest rate of single organism BSI (89.5%). Internal Medicine on the department/unit level and general wards on the ward type level registered the highest numbers of BSI across all organisms (Table 1).

**Table 1:** Characteristics of patients with Gram-negative BSI stratified by species (N = absolute numbers, % = without missing data)

	*E. coli* (N = 5412)	*Klebsiella spp.* (N = 2148)	*Enterobacter spp.* (N = 696)	*P. aeruginosa* (N = 789)	*A. baumannii* (N = 31)
Age (years), median and range	70 (18–118)	68 (20–100)	67 (20–95)	67 (18–96)	64 (27–84)
Male sex, n (%)	2926 (54.1%)	1355 (63.1%)	413 (59.3%)	520 (65.9%)	19 (61.3%)
Community onset, n (%)	3560 (65.8%)	1100 (51.2%)	283 (40.7%)	322 (40.8%)	14 (45.2%)
Single organism BSI, n (%)	4845 (89.5%)	1691 (78.7%)	489 (70.3%)	610 (77.3%)	20 (64.5%)
Department/Unit, n (%)					
Internal Medicine^1^	2845 (53.0%)	1042 (48.8%)	304 (43.9%)	294 (37.6%)	17 (54.8%)
Haematology/oncology	613 (11.4%)	210 (9.8%)	64 (9.2%)	173 (22.2%)	3 (9.7%)
Surgery^2^	978 (18.2%)	514 (24.1%)	192 (27.7%)	155 (19.8%)	4 (12.9%)
Other^3^	928 (17.3%)	371 (17.4%)	132 (19.1%)	159 (20.4%)	7 (22.6%)
Ward type, n (%)					
General ward	3972 (74.0%)	1430 (66.9%)	459 (66.3%)	475 (60.8%)	23 (74.2%)
ICU/IMC	1385 (25.8%)	705 (33.0%)	232 (33.5%)	305 (39.1%)	8 (25.8%)
Other^4^	7 (0.1%)	2 (0.1%)	1 (0.1%)	1 (0.1%)	0 (0%)

^1^Excl. Haematology/oncology

^2^Surgical services included visceral and cardiovascular surgery, neurosurgery, traumatology and orthopedic surgery

^3^Other department/units included neurology, gynecology, urology

^4^Other ward types included early rehabilitation, outpatient clinic/polyclinic, other

### Incidence rates per 1000 hospital admissions

**Fig. 1:**
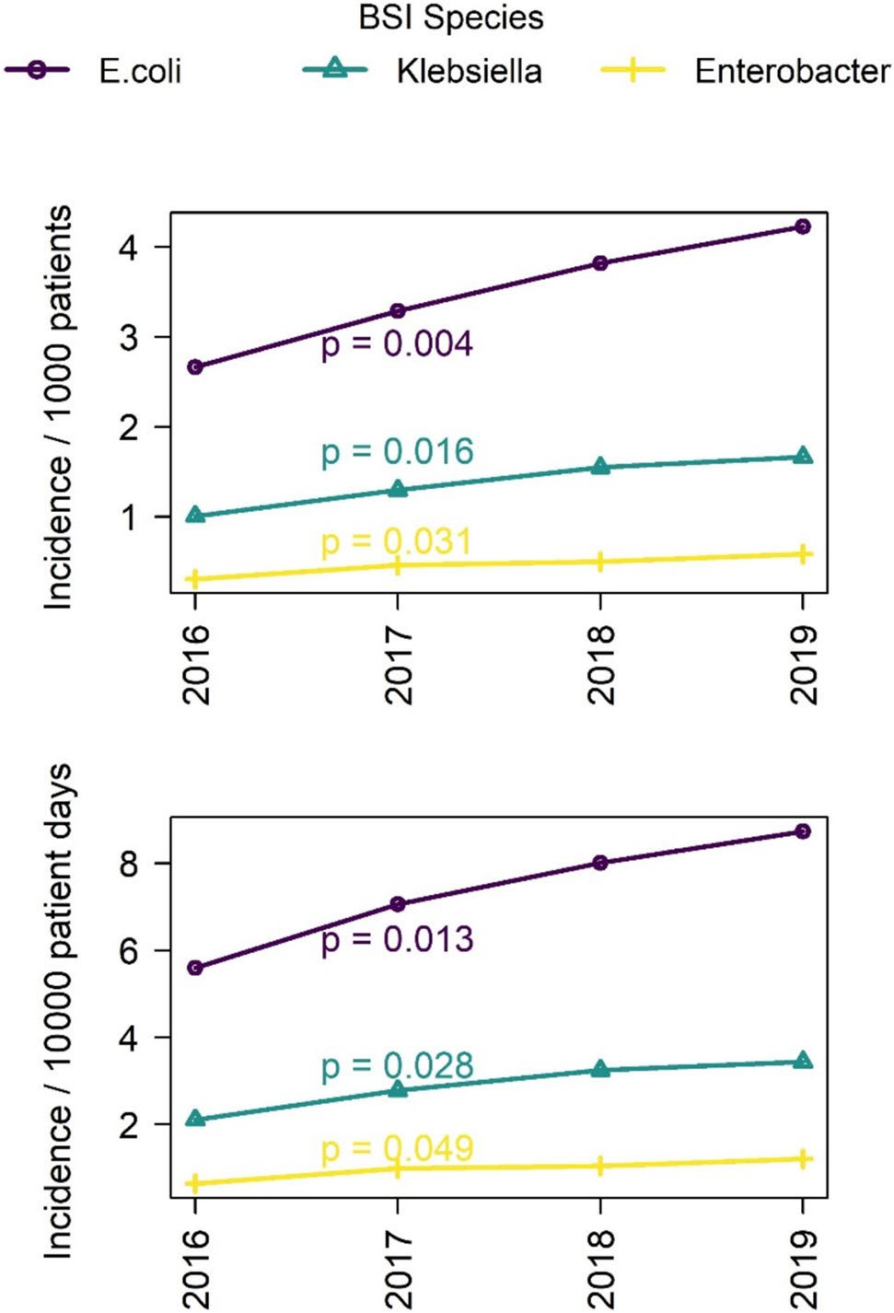
Incidence and incidence density of BSI due to *E. coli*, *Klebsiella* spp. and *Enterobacter* spp., statistical significance calculated with linear regression (p ≤ 0.05 [*]). **A** Annual trends for pooled incidence of BSI (number of BSI per 1000 admissions). **B** Annual trends for pooled incidence density of BSI (number of BSI per 10000 patient days)

Table 2 presents the incidence rates of Gram-negative BSI per 1000 admissions across various hospital departments/units, highlighting the distribution of infections. At all sites, the incidence was highest in haematology/oncology, in particular for *E. coli* BSI that ranged between 7 and 32.1 cases per 1000 admissions, compared with a range of 5 to 6.9 across centers in internal medicine (data not shown). In comparison, the general ward and ICU/IMC displayed lower incidence rates across all organisms with general ward infections peaking at 3.8 for *E. coli* and ICU/IMC at 6.9, also for *E. coli*. Incidence rates in the surgery department and other specified units were comparatively lower, with the surgery department showing an incidence rate of 2.2 for *E. coli*. Figure 1 offers a visual representation of the annual trends in the incidence and incidence density of BSI using linear regression. The annual trends for the pooled incidence of BSI (number of BSI per 1000 admissions) showed a significant increase for *E. coli*, *Klebsiella* spp. and *Enterobacter* spp. from 2016 to 2019 (Fig. 1A). The pooled incidence density of BSI (number of BSI per 10000 patient days) also increased significantly for the three organisms from 2016 to 2019 (Fig. 1B).

**Table 2:** Incidence of Gram-negative BSI (no. of BSI per 1000 admissions) across centers and different departments/units

	*E. coli* (N = 5412)	*Klebsiella spp.* (N = 2148)	*Enterobacter spp.* (N = 696)	*P. aeruginosa* (N = 789)	*A. baumannii* (N = 31)
Department/Unit					
Internal Medicine^1^	6.2	2.3	0.7	0.6	0
Haematology/oncology	13.9	4.8	1.5	3.9	0.1
Surgery^2^	2.2	1.1	0.4	0.3	0
Other^3^	3.2	1.3	0.5	0.5	0
Ward type					
General ward	3.8	1.4	0.4	0.5	0
ICU/IMC	6.9	3.5	1.2	1.5	0

^1^Excl. Haematology/oncology

^2^Surgical services included visceral and cardiovascular surgery, neurosurgery, traumatology and orthopedic surgery

^3^Other included neurology, gynecology, urology

### Overall antimicrobial resistance profiles of BSI

Table 3 outlines the annual rates of in vitro antibiotic resistance for various antibiotics across participating centers over five years (2016–2020) for various antibiotics. 3GCREB *E. coli* showed a gradual increase from 13.9% in 2016 to 14.4% in 2020, indicating a slight but continuous rise over 5 years. This trend was also seen for isolates with both when 3GCREB and is combined with ciprofloxacin resistance (CIPR) or cotrimoxazole resistance (TSXR), where rates fluctuated but generally indicated persistence or a slight reduction by 2020. In contrast, 3GCR *Klebsiella* spp. decreased considerably from 16.5% in 2016 to 11.1% in 2020. When looking on CIPR and TSXR no clear trend was observed. 3GCR *Enterobacter* spp. increased from 25% in 2016 to 38% in 2020. The combination of 3GCR with CIPR and TSXR among *Enterobacter* BSI isolates showed a decreased prevalence in 2020. More detailed information about MIC distributions for the target organisms is reported in Suppl. Tables 2 and 3.

**Table 3:** Annual rates of in vitro resistance es across centers, in % to antimicrobial agents, 3GCREB and combined resistance phenotypes over time in *E. coli*, *Klebsiella* spp. and *Enterobacter* spp. over time in the participating centers (per 100 cases with respective BSI)

	2016	2017	2018	2019	2020
*E. coli*					
PIP	11.0	9.5	9.3	8.6	8.7
CAZ	7.6	6.8	7.5	7.2	6.8
CTX	13.4	12.6	13.7	14.9	14.1
CIP	28.2	27.1	27.6	24.7	20.0
TSX	32.4	34.1	32.6	36.6	30.3
GEN	6.7	9.3	10.2	8.6	7.7
MEM	0.4	0.0	0.0	0.0	0.0
3GCREB	13.9	12.6	14	15.1	14.4
3GCREB + CIPR	9.2	9.3	10.5	10.2	8.7
3GCREB + TSXR	8	7.9	8.8	9.1	8.5
*Klebsiella* spp.					
PIP	33.3	24.5	24.6	24.5	26.0
CAZ	15.4	15.2	9.5	9.2	7.3
CTX	15.6	16	10	10.6	10.2
CIP	19.8	17.4	12.6	12.6	13.1
TSX	20.9	15.9	15.1	12	12.5
GEN	7.8	7.4	4.8	4.2	4.4
MEM	0.0	0.2	0.3	0.3	0.3
3GCREB	16.5	16.5	10.5	11.6	11.1
3GCREB + CIPR	9.9	12.3	6.4	5.9	7.9
3GCREB + TSXR	9.9	11.3	6.2	6.4	7.3
*Enterobacter* spp.					
PIP	28.0	26.9	27.1	31.8	38.8
CAZ	25.0	24.9	24.7	27.9	33.8
CTX	25	26.5	28.1	31.2	37.1
CIP	7.1	11.0	7.0	5.5	4.3
TSX	3.7	9.3	4.9	9.5	8.6
GEN	0.0	2.8	2.2	3.6	4.2
MEM	0.0	0.6	0.5	0.5	0.0
3GCREB	25	26.4	28	31.4	38
3GCREB + CIPR	3.6	5.5	5.4	4.1	2.8
3GCREB + TSXR	0	5.5	2.2	4.5	4.2

*PIP*  piperacillin, *CAZ*  ceftazidime, *CTX*  cefotaxime, *CIP*  ciprofloxacin, *TSX*  cotrimoxazole, *GEN*  gentamicin, *MEM*  meropenem, *3GCREB*  third-generation cephalosporin resistant Enterobacterales, *CIPR*  ciprofloxacin resistance, *TSXR*  cotrimoxazole resistance according to EUCAST breakpoints (v 14.0) (www.eucast.org/clinical_breakpoints)

### Clinical features and outcomes in a subgroup of *E. coli* and *Klebsiella* spp. BSI

As shown in supplementary Table 4, the mean age of patients affected by these pathogens was similar. However, a significantly higher percentage of male patients were affected by *Klebsiella* spp. infections (64.5%) compared to *E. coli* (56.4%). *E. coli* infections were more frequently community-acquired than *Klebsiella* spp. BSI (69.3% versus 47.7%, p < 0.001). There was also a difference between the two groups regarding the primary clinical focus of the infections; urogenital infections were more common in *E. coli* cases (47.9%), whereas *Klebsiella* spp. showed a more diversified clinical focus distribution, including higher rates of pulmonary infections. A slightly lower proportion of patients with *E. coli* BSI was admitted to ICU/IMC (21.1% vs 25.3% for *Klebsiella* spp.), but the difference was not statistically significant. Regarding service or specialty, *E. coli* infections were more frequently managed within internal medicine (50.2% vs. 42.9%) and, consequently less often in the surgical services (18.1% vs. 26%). With regard to HIV infection more patients were positive with *Klebsiella* spp. BSI than with *E. coli* BSI with low absolute patient numbers (2 vs. 4) (Suppl. Table 4).

Single-organism BSI were more frequently associated with *E. coli* (93.2%) than *Klebsiella* spp. (84.7%), underscoring a notable difference in the complexity of infections caused by these pathogens (p < 0.001). The case-fatality rate overall was high (18% for *E.coli* BSI, and 24% for *Klebsiella* spp. BSI), and 40% of the *E. coli* BSI patients and 38% of the *Klebsiella* spp. BSI patients were discharged with sequelae. Approx. 40% of patients with *E. coli* and 38% of patients with *Klebsiella* spp. BSI were discharged with sequelae (Suppl. Table 4).

As shown in supplementary Table 5, a high PBS (≥ 4) was the main factor associated with death (OR = 8.86, 95% CI: 2.92–26.87)—besides ICU/IMC admission (OR = 3.03, 95% CI: 1.14–8.07), and non-urinary and non-intraabdominal focus of infection. This was confirmed in a separate analysis for *E. coli* BSI, but for *Klebsiella* spp. BSI (Suppl. Tables 6 and 7). Case-fatality was higher for 3GCREB compared with third-generation cephalosporin susceptible Enterobacterales (3GCSE) both in *E. coli* BSI (22.3% vs. 17.5%) and in *Klebsiella* spp. BSI (27.5% vs 17.5%, respectively), but 3GCR was not an independent factor of lethal outcome (Suppl. Table 5).

## Discussion

Our study identified a total of 9076 patients with Gram-negative BSI over a five-year period (2016–2020) in six German university hospitals, with *E. coli* (59.6%) being the most prevalent pathogen, followed by *Klebsiella* spp. (23.7%), reflecting trends observed both nationally and across Europe [8]. *A. baumannii* BSI incidence was very low in the 5-year study period (31 cases). *E. coli* and *Klebsiella* spp. as very relevant pathogens are long known for their clinical relevance and have been recently highlighted as two of the most relevant pathogens responsible for the "burden of infection" [9], which is supported by other studies [10].

The increasing trend of AMR in our study echoes the broader situation in Germany and across Europe, where AMR remains a major public health challenge [8, 11]. Our detailed analysis contributes specific insights into the German context, offering valuable data for shaping national and regional antimicrobial stewardship and infection control policies.

Our data show rather stable 3GCR rates for *E. coli* and *Klebsiella* spp., contrasting with global concerns about the spread of AMR [12]. The incidence of carbapenem resistance remains relatively low in our study with 0.4% highest for *E. coli* and 0.3% for *Klebsiella* spp. (Table 3) being different from findings from other European studies. Nevertheless the presence of 3GCREB raises alarms, echoing the concerns highlighted by both German and European health authorities about the escalating challenge of AMR [8]. For our *E. coli* and *Klebsiella* spp. it can be assumed that 3GCREB is mostly due to ESBLs and less frequently due to AmpCs [13, 14], though specific tests for ESBL and AmpC were not part of the study design. A systematic review and meta-analysis reported a steady increase in the prevalence of ESBL-producing Enterobacterales in European countries, with significant variability between countries highlighting the emergence of community-acquired BSI caused by ESBL-producing Enterobacterales which expands the threat of these organisms beyond hospital settings, necessitating broader surveillance and containment strategies [15].

The relationship between antimicrobial resistance and clinical outcomes is a key concern in managing BSI. In our analysis the 3GCR phenotype in *E. coli* or *Klebsiella* spp. BSI was not a risk factor for adverse outcomes. These statements are central to our work and are based on the subanalysis carried out without taking into account the empirical and specific antibiotic therapy used and also without 6-month follow-up. While several studies have indicated that AMR is associated with higher mortality rates, longer hospital stays, and increased healthcare costs, mostly without taking into account specific regional differences or the impact of antimicrobial stewardship programmes. [16], other studies did not find an increased short- or long-term mortality in *E. coli* BSI either, particularly for BSI with predominance of community-acquisition like in our cohort [17]. Although less frequent, BSI due to ESBL-positive *K. pneumoniae* carried a worse prognosis in another German study including a higher in-hospital mortality [18]. A meta-analysis showed that bacteremia with ESBL-producing Enterobacterales was associated with a higher mortality compared with bacteremia due to non-ESBL producers [19]. In a BSI study due to Enterobacterales at ten European hospitals the 3GCR phenotype increased the hazard of death compared with third-generation cephalosporin susceptibility (adjusted hazard ratio 1.63; 95% CI 1.13–2.35) [20]. An analysis that included non-BSI infections, the estimated number of attributable deaths due to infections with 3GCREB *E. coli* and *Klebsiella* spp. increased more than four-fold [21]. However, in patients with strict community-onset bacteremia, this association is less well established, with studies producing conflicting results [22–24]. Differences in mortality might, however, partly be explained by heterogeneous studies, including/excluding polymicrobial bacteremia, different patient features, different circulating regional strains and treatment strategies. According to some work, the increased risk of death has been related to delays in the initiation of effective antimicrobial therapy [25]. Nevertheless, the impact of an adequate empirical antibiotic treatment is of course high and has to be considered [26]. For example, others found a higher crude 14 day mortality for bacteraemic patients due to ESBL producers, but the association disappeared when adjusting for inappropriate antibiotic therapy [24]. Data on empirical and targeted antimicrobial treatment were not available for the present analysis and has to be investigated by a different study design (case–control study), so that the interpretation has to be drawn with caution.

In other works, further risk factors of ESBL BSI were identified like obstructive urinary tract disease, previous surgical history and the prior use of a cephalosporin antibiotic within 3 months [27]. Our findings further demonstrated that patients with a higher PBS faced worse outcomes, a relationship that has been well-documented. Specifically, we observed that a score ≥ 4 was associated with significantly increased odds of death (OR = 8.86, 95% CI: 2.92–26.87) but not discharge with sequelae, underscoring the utility of this score in predicting BSI mortality and morbidity.

The high incidence of BSI observed in haematology/oncology departments underscores the unique vulnerability of this patient population, primarily due to immunosuppression caused by both the malignancies themselves and the intensive chemotherapy regimens required for treatment. Interestingly resistance rates to piperacillin also slightly decreased over time (Table 3) without notable changes in therapeutic regimens to our knowledge. Patients in haematology/oncology were in general more susceptible to invasive infections, including those caused by multi-drug resistant organisms, due to their compromised immune systems and frequent use of invasive devices, such as central venous catheters [28–30].

Strengths of the current study include the prospective study design, the stratification by department and the high number of cases which allows to highlight distinct epidemiological aspects of Gram-negative BSI in ICU and non-ICU settings over the study period. The multicentre analysis allows finding general trends over several years. Our study is not without limitations. The study did not start at the beginning of 2016, but Q4 2016 and ended in Q2 2020, i.e. the analysis does not include complete years which may lead to uncertainties of measurements. A challenge in evaluating the datasets was merging the data from two different databases (R-NET and BLOOMY cohort studies). Additional clinical data, e.g., information on empiric and definitive as well as appropriate antibiotic therapy, is missing. This is an important factor, especially in infections with multidrug-resistant organisms, where delayed or inappropriate therapy can lead to worse outcomes. Due to the study design this data is not available and can therefore not be included in the analysis. Our data exclusively reflect the epidemiological scenario in tertiary care hospitals which may overestimate the incidence of AMR compared to hospitals of primary or secondary care level and limits generalizability of our data.

## Conclusions

In conclusion, our study provides a comprehensive overview of the epidemiology, antimicrobial resistance patterns, and clinical outcomes of Gram-negative BSI in German university hospitals. By situating our findings within the broader context of German and European data, we contribute valuable insights into the challenges and opportunities for improving BSI management. These data underscores the critical need for ongoing surveillance and more pathogen-related clinical data for Germany including infection control and treatment strategies to address the pressing public health threat posed by AMR. Future research should aim to include a broader range of hospital types and regions to capture a more comprehensive picture of BSI and AMR in Germany and Europe. Research should focus on longitudinal studies to track the evolution of resistance patterns and explore the impact of novel antimicrobial agents and stewardship interventions on BSI outcomes.

## Supplementary Information

Below is the link to the electronic supplementary material.

Supplementary Material 1.

Supplementary Material 2.

Supplementary Material 3.

Supplementary Material 4.

Supplementary Material 5.

Supplementary Material 6.

Supplementary Material 7.

## Data Availability

No datasets were generated or analysed during the current study.
