# Supplementary material for: Gram-negative bloodstream infections in six German university hospitals, 2016–2020: clinical and microbiological features
Source: Infection. 2024 Nov 25;53(2):625–33. doi: 10.1007/s15010-024-02430-7 (PMC11971176; doi:10.1007/s15010-024-02430-7)
Supplement: Supplementary file 1 — Supplementary Material 1. [file 15010_2024_2430_MOESM1_ESM.docx]

**Suppl. table 1**. Species differentiation among *Klebsiella* spp. and *Enterobacter* spp. BSI

isolates

|  | *Klebsiella* spp. (N=2,148) | *Enterobacter* spp. (N=696) |
| --- | --- | --- |
|  |  |  |
| *Klebsiella pneumoniae* | 1506 (70.1%) | - |
| *Klebsiella oxytoca* | 441 (20.5%) | - |
| *Klebsiella aerogenes* | 95 (4.4%) | - |
| Other *Klebsiella* spp. | 60 (2.8%) | - |
| *Enterobacter cloacae* complex | - | 658 (94.5%) |
| Other *Enterobacter* spp. | - | 38 (5.5%) |
