# Supplementary material for: Gram-negative bloodstream infections in six German university hospitals, 2016–2020: clinical and microbiological features
Source: Infection. 2024 Nov 25;53(2):625–33. doi: 10.1007/s15010-024-02430-7 (PMC11971176; doi:10.1007/s15010-024-02430-7)
Supplement: Supplementary file 2 — Supplementary Material 2. [file 15010_2024_2430_MOESM2_ESM.docx]

**Suppl. table 2.** MIC50 and MIC90 values (mg/L) and antimicrobial susceptibilities of *E. coli*, *Klebsiella* spp. and *Enterobacter* spp. BSI isolates

|  | ***E. coli* (N=5412)** | | | | | ***Klebsiella* spp. (N=2148)** | | | | | ***Enterobacter* spp. (N=696)** | | | | |
| --- | --- | --- | --- | --- | --- | --- | --- | --- | --- | --- | --- | --- | --- | --- | --- |
| Antimicrobial agent | MIC_50_ | MIC_90_ | MIC range | %S | %R | MIC_50_ | MIC_90_ | MIC range | %S | %R | MIC_50_ | MIC_90_ | MIC range | %S | %R |
| Cefotaxime | ≤1 | ≥64 | ≤1 to ≥64 | 86.2 | 13.8 | ≤1 | 8 | ≤1 to ≥64 | 88.1 | 11.9 | ≤1 | ≥64 | ≤1 to ≥64 | 70.5 | 29.5 |
| Ceftazidime | ≤1 | 4 | ≤1 to ≥64 | 92.8 | 7.2 | ≤1 | 8 | ≤1 to ≥64 | 89.3 | 10.7 | ≤1 | 64 | ≤1 to ≥64 | 73.3 | 26.7 |
| Ciprofloxacin | ≤0.25 | ≥4 | ≤0.25 to ≥4 | 74.5 | 25.5 | ≤0.25 | 1 | ≤0.25 to ≥4 | 85.9 | 14.1 | ≤0.25 | ≤0.25 | ≤0.25 to ≥4 | 92.7 | 7.3 |
| Cotrimoxazol | ≤1 | ≥16 | ≤1 to ≥16 | 66.2 | 33.8 | ≤1 | ≥16 | ≤1 to ≥16 | 85.8 | 14.2 | ≤1 | ≤1 | ≤1 to ≥16 | 92.1 | 7.9 |
| Imipenem | ≤0.25 | ≤0.25 | ≤0.25 to ≥16 | 100 | - | ≤0.25 | ≤0.25 | ≤0.25 to ≥16 | 99.5 | 0.5 | ≤0.25 | 1 | ≤0.25 to ≥16 | 99.6 | 0.4 |
| Meropenem | ≤0.25 | ≤0.25 | ≤0.25 to ≥16 | 100 | - | ≤0.25 | ≤0.25 | ≤0.25 to ≥16 | 99.7 | 0.3 | ≤0.25 | ≤0.25 | ≤0.25 to ≥16 | 99.6 | 0.4 |
| Piperacillin | ≤4 | 8 | ≤4 to ≥128 | 90.9 | 9.1 | ≤4 | ≥128 | ≤4 to ≥128 | 74.9 | 25.1 | ≤4 | ≥128 | ≤4 to ≥128 | 70.1 | 29.9 |
| Gentamicin | ≤1 | ≤1 | ≤1 to ≥16 | 91.0 | 9.0 | ≤1 | ≤1 | ≤1 to ≥16 | 94.7 | 5.3 | ≤1 | ≤1 | ≤1 to ≥16 | 97.1 | 2.9 |
