# Supplementary material for: Gram-negative bloodstream infections in six German university hospitals, 2016–2020: clinical and microbiological features
Source: Infection. 2024 Nov 25;53(2):625–33. doi: 10.1007/s15010-024-02430-7 (PMC11971176; doi:10.1007/s15010-024-02430-7)
Supplement: Supplementary file 3 — Supplementary Material 3. [file 15010_2024_2430_MOESM3_ESM.docx]

**Suppl. table 3.** MIC50 and MIC90 values and antimicrobial susceptibilities of *P. aeruginosa* and *A. baumannii* BSI isolates

|  | ***P. aeruginosa* (N=789)** | | | | | ***A. baumannii* (N=31)** | | | | |
| --- | --- | --- | --- | --- | --- | --- | --- | --- | --- | --- |
| Antimicrobial agent | MIC_50_ | MIC_90_ | MIC range | %S | %R | MIC_50_ | MIC_90_ | MIC range | %S | %R |
| Ceftazidime | 4 | 16 | ≤1 to ≥64 | 86.7 | 13.3 | - | - | - | - | - |
| Ciprofloxacin | ≤0.25 | 2 | ≤0.25 to ≥4 | 82.4 | 17.6 | ≤0.25 | ≥4 | ≤0.25 to ≥4 | 83.9 | 16.1 |
| Cotrimoxazol | - | - | - | - | - | ≤1 | ≥4 | ≤1 to ≥16 | 83.9 | 16.1 |
| Imipenem | 2 | ≥16 | ≤0.25 to ≥16 | 83.2 | 16.8 | ≤0.25 | ≤0.25 | ≤0.25 to ≥16 | 96.3 | 3.7 |
| Meropenem | ≤0.25 | 8 | ≤0.25 to ≥16 | 90.7 | 9.3 | ≤0.25 | 0.5 | ≤0.25 to ≥16 | 95.8 | 4.2 |
| Piperacillin | 8 | ≥128 | ≤4 to ≥128 | 80.7 | 19.3 | - | - | - | - | - |
