# Supplementary material for: Gram-negative bloodstream infections in six German university hospitals, 2016–2020: clinical and microbiological features
Source: Infection. 2024 Nov 25;53(2):625–33. doi: 10.1007/s15010-024-02430-7 (PMC11971176; doi:10.1007/s15010-024-02430-7)
Supplement: Supplementary file 4 — Supplementary Material 4. [file 15010_2024_2430_MOESM4_ESM.docx]

**Suppl. table 4.** Characteristics and clinical correlates of patients with BSI due to *E. coli* and *Klebsiella* spp. (subgroup analysis).

|  | ***E. coli* (N=913)** | ***Klebsiella* spp. (N=313)** | **p-value** |
| --- | --- | --- | --- |
| Mean age, years (SD) | 67.2 (14.2) | 66.3 (13.6) | ns |
| Male sex, n (%) | 515 (56.4%) | 202 (64.5%) | 0.01 |
| PBS ≥4 at onset of BSI | 102 (12.0%) | 45 (15.9%) | ns |
| Previous stay in long-term care facilities within 3 months | 46 (5.0%) | 10 (3.2%) | ns |
| Previous stay in rehabilitation centre within 3 months | 29 (3.2%) | 10 (3.2%) | ns |
| Previous hospital admission within 3 months | 326 (35.7%) | 93 (29.7%) | ns |
| Community-acquired infection | 616 (69.3%) | 146 (47.7%) | <0.001 |
| Clinical focus |  |  |  |
| Urogenital | 419 (47.9%) | 82 (28.1%) | <0.001 |
| Abdominal | 209 (23.9%) | 81 (27.7%) |  |
| Pulmonary | 88 (10.1%) | 51 (17.5%) |  |
| Other/unknown | 158 (18.1%) | 78 (26.7%) |  |
| Underlying diseases/comorbidity |  |  |  |
| Chronic Liver disease | 63 (7.1%) | 24 (7.9%) | ns |
| Solid tumor | 422 (47.6%) | 156 (50.6%) | ns |
| Metastatic solid tumor | 145 (17.2%) | 64 (21.7%) | ns |
| Leukemia | 52 (9.1%) | 17 (8.81) | ns |
| Lymphoma | 48 (8.4%) | 18 (9.3%) | ns |
| HIV infection | 2 (0.3%) | 4 (1.6%) | 0.04 |
| Chronic bowel disease | 40 (4.8%) | 17 (5.8%) | ns |
| Ward type at onset of BSI |  |  |  |
| ICU/IMC | 192 (21.1%) | 79 (25.3%) | ns |
| General ward | 719 (78.9%) | 233 (74.7%) |  |
| Department/Unit |  |  |  |
| Internal Medicine^1^ | 457 (50.2%) | 134 (42.9%) | 0.02 |
| Haematology/oncology | 123 (13.5%) | 44 (14.1%) |  |
| Surgery^2^ | 165 (18.1%) | 81 (26.0%) |  |
| Other^3^ | 166 (18.2%) | 53 (17.0%) |  |
| Single-organism BSI, n (%) | 851 (93.2%) | 265 (84.7%) | <0.001 |
| Status of discharge |  |  |  |
| Full recovery | 382 (42.3%) | 120 (38.5%) | ns |
| Sequelae (impaired) | 359 (39.7%) | 117 (37.5%) |  |
| Death | 163 (18%) | 75 (24%) |  |

^1^excl. Haematology/oncology

^2^Surgical services included visceral and cardiovascular surgery, neurosurgery, traumatology and orthopedic surgery

^3^Other included neurology, gynecology, urology
