# Supplementary material for: Gram-negative bloodstream infections in six German university hospitals, 2016–2020: clinical and microbiological features
Source: Infection. 2024 Nov 25;53(2):625–33. doi: 10.1007/s15010-024-02430-7 (PMC11971176; doi:10.1007/s15010-024-02430-7)
Supplement: Supplementary file 5 — Supplementary Material 5. [file 15010_2024_2430_MOESM5_ESM.docx]

**Suppl. table 5** Overall risk factors for discharge with sequelae (impaired) or death compared to full recovery by multinomial log-linear regression analysis

|  | **Adjusted OR of sequelae (95% CI)** | **Adjusted OR of death (95% CI)** |
| --- | --- | --- |
| Sex: F vs. M | 1.27 (0.77, 2.08) | 1.48 (0.73, 3.02) |
| Liver disease | 0.97 (0.37, 2.54) | 3.12 (0.94, 10.38) |
| Solid tumor | 0.61 (0.31, 1.21) | 0.88 (0.33, 2.3) |
| Advanced metastatic tumor | 2.03 (0.93, 4.42) | 1.44 (0.47, 4.42) |
| Leukemia | 0.79 (0.31, 2.02) | 0.87 (0.26, 2.94) |
| Lymphoma | 0.72 (0.27, 1.95) | 0.5 (0.11, 2.36) |
| HIV | NA | 16.02 (0.64, 403.02) |
| Chronic bowel disease | 0.3 (0.06, 1.42) | 1.83 (0.49, 6.8) |
| Ward type: ICU/IMC vs. general | 1.53 (0.7, 3.35) | 3.03 (1.14, 8.07) |
| Age | 1.01 (0.99, 1.03) | 1.05 (1.02, 1.08) |
| Species: *Klebsiella* spp. vs. *E. coli* | 1.14 (0.64, 2.05) | 0.67 (0.28, 1.64) |
| Mode of acquisition: hospital-acquired vs. community-acquired | 0.67 (0.39, 1.15) | 1.04 (0.48, 2.24) |
| 3GCREB vs. 3GCSE | 0.94 (0.42, 2.1) | 0.83 (0.27, 2.51) |
| Ciprofloxacin: R vs. S | 1.55 (0.78, 3.1) | 1.51 (0.59, 3.91) |
| Cotrimoxazole: R vs. S | 0.87 (0.47, 1.59) | 1.28 (0.54, 3.06) |
| Gentamicin: R vs. S | 1.44 (0.49, 4.22) | 1.74 (0.42, 7.23) |
| Piperacillin: R vs. S | 1.27 (0.62, 2.61) | 1.45 (0.54, 3.87) |
| PBS ≥4 vs. <4 | 2.54 (0.87, 7.4) | 8.86 (2.92, 26.87) |
| Focus of infection: |  |  |
| abdominal | 0.82 (0.44, 1.51) | 2.21 (0.87, 5.63) |
| other/unknown | 1.45 (0.73, 2.88) | 3.47 (1.25, 9.64) |
| pulmonary/respiratory | 1.62 (0.59, 4.43) | 4.2 (1.13, 15.65) |
