# Supplementary material for: Gram-negative bloodstream infections in six German university hospitals, 2016–2020: clinical and microbiological features
Source: Infection. 2024 Nov 25;53(2):625–33. doi: 10.1007/s15010-024-02430-7 (PMC11971176; doi:10.1007/s15010-024-02430-7)
Supplement: Supplementary file 6 — Supplementary Material 6. [file 15010_2024_2430_MOESM6_ESM.docx]

**Suppl. table 6** *E. coli* risk factors for discharge with sequelae (impaired) or death compared to full recovery by multinomial log-linear regression analysis

|  | **Adjusted OR of sequelae (95% CI)** | **Adjusted OR of death (95% CI)** |
| --- | --- | --- |
| Sex: F vs. M | 1.23 (0.68, 2.22) | 1.26 (0.52, 3.05) |
| Liver disease | 1.65 (0.54, 5.06) | 4.49 (1.01, 19.92) |
| Solid tumor | 0.97 (0.42, 2.23) | 0.88 (0.26, 2.99) |
| Advanced metastatic tumor | 1.81 (0.72, 4.56) | 0.63 (0.13, 3.11) |
| Leukemia | 0.86 (0.29, 2.61) | 1.14 (0.24, 5.35) |
| Lymphoma | 0.97 (0.29, 3.26) | 0.51 (0.06, 3.98) |
| HIV | 1 (1,1) | 1 (1, 1) |
| Chronic bowel disease | 0.17 (0.02, 1.45) | 1.74 (0.36, 8.43) |
| Ward type: ICU/IMC vs. general | 2.23 (0.86, 5.81) | 1.24 (0.32, 4.7) |
| Age | 1.01 (0.99, 1.03) | **1.04 (1.01, 1.08)** |
| Mode of acquisition: hospital-acquired vs. community-acquired | **0.49 (0.25, 0.96)** | 0.55 (0.21, 1.43) |
| 3GCREB vs. 3GCSE | 1.23 (0.47, 3.2) | 1.35 (0.33, 5.44) |
| Ciprofloxacin: R vs. S | 1.63 (0.77, 3.48) | 1.69 (0.58, 4.93) |
| Cotrimoxazole: R vs. S | 0.83 (0.42, 1.63) | 1.09 (0.38, 3.07) |
| Gentamicin: R vs. S | 1.57 (0.49, 5.05) | 2.58 (0.51, 12.91) |
| Piperacillin: R vs. S | 1.33 (0.54, 3.32) | 1.8 (0.55, 5.94) |
| PBS ≥4 vs. <4 | 1.04 (0.26, 4.24) | **16.76 (4.42, 63.52)** |
| Focus of infection vs. urogenital: |  |  |
| abdominal | 0.91 (0.45, 1.87) | 2.89 (0.9, 9.25) |
| other/unknown | 1.96 (0.84, 4.55) | **7.4 (2.07, 26.4)** |
| pulmonary/respiratory | 1.32 (0.35, 4.93) | **14.53 (3.06, 69.06)** |
