# Supplementary material for: Gram-negative bloodstream infections in six German university hospitals, 2016–2020: clinical and microbiological features
Source: Infection. 2024 Nov 25;53(2):625–33. doi: 10.1007/s15010-024-02430-7 (PMC11971176; doi:10.1007/s15010-024-02430-7)
Supplement: Supplementary file 7 — Supplementary Material 7. [file 15010_2024_2430_MOESM7_ESM.docx]

**Suppl. table 7** *Klebsiella* spp. risk factors for discharge with sequelae (impaired) or death compared to full recovery by multinomial log-linear regression analysis

|  | **Adjusted OR of sequelae (95% CI)** | **Adjusted OR of death (95% CI)** |
| --- | --- | --- |
| Sex: F vs. M | 1.65 (0.47, 5.84) | 3.2 (0.41, 24.87) |
| Liver disease | NA | 0.48 (0, 127.57) |
| Solid tumor | 0.22 (0.05, 1.02) | 1.03 (0.09, 11.95) |
| Advanced metastatic tumor | 3.39 (0.44, 25.93) | 8.15 (0.44, 151.86) |
| Leukemia | 0.65 (0.03, 13.29) | 0.5 (0.01, 44.72) |
| Lymphoma | 0.21 (0.02, 2.75) | 0.81 (0.03, 22.07) |
| HIV | NA | 1.41 (0.02, 105.9) |
| Chronic bowel disease | 2.82 (0.13, 63.32) | 7.99 (0.16, 388.08) |
| Ward type: ICU/IMC vs. general | 0.49 (0.06, 4.18) | 8.32 (0.6, 116.17) |
| Age | 1.03 (0.98, 1.07) | 1.08 (0.97, 1.19) |
| Mode of acquisition: hospital-acquired vs. community-acquired | 0.78 (0.19, 3.09) | **16.21 (1.21, 217.76)** |
| 3GCREB vs. 3GCSE | 0.21 (0.01, 3) | 0.03 (0, 2.03) |
| Ciprofloxacin: R vs. S | 0.19 (0, 7.68) | NA |
| Cotrimoxazole: R vs. S | 1.53 (0.11, 21.22) | 7.93 (0.18, 343.73) |
| Gentamicin: R vs. S | NA | NA |
| Piperacillin: R vs. S | 1.51 (0.25, 9.03) | 3.06 (0.16, 57.76) |
| PBS ≥4 vs. <4 | 15.28 (0.61, 381.93) | 4.63 (0.1, 218.85) |
| Focus of infection vs. urogenital: |  |  |
| abdominal | 0.27 (0.05, 1.35) | 0.82 (0.04, 15.63) |
| other/unknown | 0.45 (0.08, 2.57) | 0.37 (0.02, 7.85) |
| pulmonary/respiratory | 0.89 (0.07, 11.53) | 0.35 (0.01, 17.59) |
